# The influence of anaesthesia on cancer growth

**DOI:** 10.2478/raon-2024-0012

**Published:** 2024-02-21

**Authors:** Iztok Potocnik, Milena Kerin-Povsic, Jasmina Markovic-Bozic

**Affiliations:** Department of Anaesthesiology and Intensive Care, Institute of Oncology Ljubljana, Ljubljana, Slovenia; Faculty of Medicine, University of Ljubljana, Ljubljana, Slovenia; Department of Anaesthesiology and Surgical Intensive Therapy, University Medical Centre Ljubljana, Ljubljana, Slovenia

**Keywords:** cancer growth, anaesthesia, inflammation

## Abstract

**Background:**

Oncological patients make up a large proportion of all surgical patients. Through its influence on the patient’s inflammatory and immune system, the choice of anaesthetic technique has an indirect impact on the health of the individual patient and on public health. Both the specific and the non-specific immune system have a major influence on the recurrence of carcinomas. The pathophysiological basis for growth and metastasis after surgery is the physiological response to stress. Inflammation is the organism’s universal response to stress. Anaesthetics and adjuvants influence perioperative inflammation in different ways and have an indirect effect on tumour growth and metastasis. *In vitro* studies have shown how individual anaesthetics influence the growth and spread of cancer, but clinical studies have not confirmed these results. Nevertheless, it is advisable to use an anaesthetic that has shown lesser effect on the growth of cancer cells *in vitro*.

**Conclusions:**

In this review, we focus on the area of the effects of anaesthesia on tumour growth. The field is still relatively unexplored, there are only few clinical prospective studies and their results are controversial. Based on the review of new research findings we report on recommendations about anaesthetics and anaesthetic techniques that might be preferable for oncological surgical procedures.

## Introduction

Perioperative morbidity and mortality have decreased over time due to the use of modern anaesthesia and surgical techniques. The question arises as to how we can influence long-term morbidity and mortality in cancer patients. Published studies have shown that an appropriate anaesthetic technique (AT) can influence the recurrence and spread of the disease.^1,2^ Oncological patients make up a large proportion of all surgical patients, and their number increases by more than 25% every five years. Two thirds of all cancer patients require at least one operation during treatment. Therefore, the choice of AT has an indirect impact on the health of the individual patient and on public health.^1,2^

Metastases develop because cancer cells evade the immune system, multiply, and spread to other tissues and organs.^3^ It has been shown that anaesthesia influences the spread of cancer through the immune system.^1,2^ Both specific and non-specific immune systems have a major influence on metastasis.^1,2^ During the perioperative period, the organism is exposed to many processes that can affect the metastasis. The most important of these are inflammation, anaesthetics, hypothermia and the transfusion of blood products.^1,2,3^

## Pathophysiology of metastasis

The pathophysiological basis for the growth and metastasis of carcinomas after surgery is the reaction to stress. The universal reaction of the organism to stress is inflammation. The organism reacts to all harmful stimuli with inflammation. During an operation, both the systemic inflammatory reaction and the ischaemia/reperfusion reaction are triggered.^4^ In addition, severe tissue damage occurs, which is also a cause of the stress reaction and inflammation. Inflammatory factors such as interleukins (ILs) and prostaglandins (PGs) are released into the bloodstream as a result of the non-specific inflammatory response. To a certain extent, they have the task of protecting the organism from harmful stimuli, but if the reaction is too strong, additional tissue damage occurs.

When inflammation escalates a vicious circle may be triggered. The most important inflammatory factors that are released and influence the growth of tumour cells are interleukin-6 (IL-6) and prostaglandin E2 (PGE2).^5^ These factors influence the reduced activity of natural killer cells, so that cellular immunity is weakened, and the tumour cells can evade the immune system and multiply. As a result of immunosuppression, certain hormones (catecholamines, PGs and growth factors) are released, which also influence the growth and metastasis of carcinomas. Tumour cells have mechanisms to increase their insensitivity to hypoxia. Due to tissue hypoxia, certain genes are expressed in tumour cells. Hypoxia inducible factor 1-alpha (HIF-1α) is released, which promotes angiogenesis, proliferation, and metastasis. High HIF-1α levels are a predictive factor for long-term morbidity and mortality due to postoperative carcinoma growth.^6^

### The impact of inflammation on metastasis

Inflammation is a universal physiological defence reaction of the organism that protects the body from harmful factors. It is triggered by the activation of the immune system and causes the elimination of harmful stimuli, prevents the spread of damage and repairs the affected tissue. It involves several reactions: vascular reaction (vasodilatation, exudation), cellular reaction (migration, adhesion, phagocytosis, degranulation) and connective tissue reaction (matrix formation, repair, angiogenesis).^4,6,7^ A distinction is made between non-specific and specific immunity: non-specific immunity is characterised by various cascade reactions and the production of inflammatory factors such as pros-taglandins and cytokines. The product of specific immunity are antibodies that are directed precisely against a specific harmful stimulus such as carcinoma cells. Cellular immunity also includes natural killer cells, which ensure the death of harmful cells (tumour cells, bacteria, blood cells in transfusion derivatives).^5,6^ Both forms of specific immunity function and communicate with each other via signalling molecules. A harmful cell labelled with antibodies is easy prey for the natural killer cells. The inflammatory reaction must be precisely regulated.^4,6,7^ An excessive inflammatory response also damages the body’s own tissue and causes postoperative complications. An excessive reaction is referred to as a systemic inflammatory response (SIRS).^7^

An inflammatory reaction is also triggered by tissue damage during the operation.^8^ Inflammation may promote the postoperative growth of any residual tumour and progression of metastasis.^4,6^ Therefore, the least possible invasive surgical technique should be used. There are three harmful perioperative reactions triggered by inflammation.^8,9^ The first harmful reaction is SIRS. The inflammatory event involves the entire organism. Many cytokines are released because their regulatory level is disturbed.^7,8,9,10,11^ In severe inflammation, SIRS can lead to organ dysfunction and organ failure. SIRS complications include acute lung injury (ALI), acute renal failure (ARF), shock and multiple organ failure (MOF).^10,11^

The second harmful reaction is the ischaemia/reperfusion reaction. When ischaemic tissue is reperfused, large amounts of reactive oxygen species (ROS) are released. If they are not neutralised and removed, they can cause tissue damage. The enzyme xanthine oxidase (XOX) plays an important role in this reaction. During ischaemia, it is formed in large quantities by the enzyme xanthine dehydrogenase (XDH) and breaks down purines. XOX remains inactive until sufficient oxygen is available. This happens when the tissue is supplied with blood again.^7,8,9,10,11^ In addition, during ischaemia there is a decrease in the regeneration of adenosine triphosphate (ATP) from adenosine diphosphate (ADP). Due to the lack of oxygen, ADP is also reduced to adenosine monophosphate (AMP) in order to generate additional energy.^7^ After reperfusion and replenishment of the tissue with oxygen, XOX is activated, and part of the AMP is degraded to uric acid. During this process, electrons are released and transferred to oxygen to form ROS. If the ROS scavengers are unable to remove these, nearby cells are damaged, and an inflammatory reaction is triggered. It is initially localised, but if severe enough, it leads to SIRS.^7^

The third adverse perioperative reaction is called acute lung injury (ALI) and acute respiratory distress syndrome (ARDS). ARDS leads to cytokine release, damage to the pulmonary vascular endothelium, decreased surfactant production and alveolar surface tension, fluid accumulation and fibrosis. The mortality rate for ARDS is 20–50%.^7,8,9,10,11^

### The effect of anaesthetics and anaesthetic technique on inflammation and metastasis

The choice of anaesthetic and adjuvants primarily influences perioperative inflammation in various ways and has indirect effects on tumour growth and metastasis.^12^

Rational anaesthesia management has a major influence on the long-term surgical outcome.^4^ Anaesthetics affect the non-specific and specific inflammatory response, the immune cascades and consequently the production of cytokines and the function of inflammatory cells.^13^ For example, propofol increases the number of killer cells but reduces their cytotoxic activity, while sevoflurane increases the number of killer cells but reduces the number and activity of other immune cell types such as CD4 T-helper and CD-8 cytotoxic T-lymphocytes. The overall effect on the immune system and inflammation may depend on many factors, including the specific combination and dose of an-aesthetic agents used.^13^

A single agent lowers the level of some cytokines and increases the level of others. Some cytokines are pro-inflammatory (TNFα, IL-1, IL-6, IL-8), while others are anti-inflammatory (IL-10). This further complicates the effect of cytokines. Studies have shown that cytokine levels in the blood increase immediately after induction of anaesthesia and even before surgery.^14^ Opioids reduce the inflammatory response because they reduce intracellular cyclic AMP, which is an important factor in stimulating IL-6 synthesis.^15^ In addition, neutrophils have opioid receptors on their membrane that inhibit their function.^16^

Pain also alters the immune response by increasing the number of activated lymphocytes and decreasing the number of inhibitory T cells and T helper cells. Inflammatory processes are particularly strongly activated in chronic pain.^17,18^

Studies have shown that intravenous anaesthetics stimulate inflammatory cells to produce cytokines.^19^ Intravenous anaesthetics inhibit the polarisation and chemotaxis of neutrophils to a greater extent than volatile anaesthetics (VA).^20^ Anaesthetics also influence proliferation, lymphocyte count and perioperative immunoglobulin levels in the blood.^21,22^ In addition to the choice of anaesthetic, different regional techniques (epidural, paravertebral anaesthesia) also influence perioperative inflammation depending on the anaesthetics used.^23,24^

Transfusion of blood derivatives reduces the number of T-cytotoxic leukocytes, TNF production and macrophage chemotaxis.^25^

Finally, the central nervous system also has an effect on perioperative stress and the immune response, which is the subject of psychoneuroimmunology.^26,27^ Thoughts and emotions also influence the immune system via centres in the brain. The hypothalamus plays a central role because it influences the sympathetic nervous system and the hypothalamic-pituitary-adrenal axis by altering catecholamine levels, corticosteroids, and opioids in the body.^27^ The concentration of growth hormone and prolactin in the blood also changes.^28^ All these processes have a significant influence on the function of the immune system. The immune system is inhibited and weakened in a stressful situation.^29^

There are not many clinical, randomised studies that have investigated the direct influence of AT on tumour growth and metastasis after surgery. The results are often controversial. The studies published in recent years have not shown any advantages of different AT such as regional, general, or combined anaesthesia.^30,31,32,33^

VA modulate the inflammatory response and have a positive anti-inflammatory effect. ^34,35^ However, it is not clear whether this also has a negative effect on tumour cells. There are observations that they have a pro-inflammatory effect and therefore accelerate metastasis. The molecular mechanism of this process is not known.^36^ *In vitro*, they have shown a mild anti-inflammatory and thus protective effect, while increased levels of HIF-1α have been observed *in vivo*.^4,7^ VA are thought to cause chemoresistance and attenuate the effect of adjuvant chemotherapy.^1,2^ *In vitro*, sevoflurane has been shown to promote inflammation via the nuclear factor kappa B (NF-κB) pathway.^37^

Propofol is known to have an anti-inflammatory effect, particularly in the central nervous system, where it prevents perioperative neuroinflammation.^35,38^ Propofol acts in the cell nucleus and influences the formation of NF-κB. *In vitro* studies have also shown an effect on the transcription of ribonucleic acid (RNA) as well as anti-inflammatory and antioxidant effects. The antitumour effect of propofol has not yet been confirmed with certainty, but it lowers HIF-1α levels.^1,2,3^ However, in triple-negative breast cancer cell lines, propofol increased the antitumour effect of doxorubicin and paclitaxel.^39^ However, clinical studies have shown very controversial results.^39^

According to published studies, ketamine and thiopental have a major impact on inflammation. They influence the function of the immune system by inhibiting NK cells.^1,2,3^ Ketamine also increases the level of anti-apoptotic protein.^2,3^

Anaesthesiologist is faced with the dilemma of whether to anaesthetise a carcinoma patient with total intravenous anaesthesia (TIVA) or with volatile induced and maintained anaesthesia (VIMA). Several studies have confirmed the anti-inflammatory effect of VA. In cardiac surgery, pre- and post-conditioning are used due to the proven anti-inflammatory and tissue-protective effect.^40^ The positive effect of sevoflurane has also been demonstrated in liver surgery, where a strong inflammatory reaction is expected.^41,42^ It is also frequently used in intensive care medicine to sedate patients. It has been shown to have positive effects on the systemic inflammatory response of the organism and works very well in ARDS.^43^ It is also used in lung surgery. During lung surgery, several reactions are triggered that lead to an excessive inflammatory response. Perioperative unilateral lung ventilation triggers an ischaemia/reperfusion reaction, which can cause additional damage to the lungs already mechanically damaged by the operation. Sevoflurane reduces the concentration of pro-inflammatory factors. Therefore, lung damage is also reduced, and fewer postoperative complications occur.^9,44^ Other studies have shown the pro-inflammatory effect of VA.^45,46^ From this it could be concluded that they cause the progression of cancer, but clinical studies have not confirmed this with certainty.

A recent meta-analysis of TIVA versus VA showed that 7,866 patients with breast, oesophageal or non-small lung cancer had improved recurrence-free survival after VIMA. In addition, studies that included 18,778 patients showed that overall survival was longer after VIMA than after TIVA.^47^ However, there were no differences between the two techniques in terms of the presence of circulating tumour cells in breast cancer patients.^48^ Furthermore, there were no effects on immune cells and cancer-regulating factors between the two AT in colorectal cancer surgery.^45^

The use of regional anaesthesia indirectly reduces the progression of cancer by decreasing the neuroendocrine response to surgery and reducing the use of opioids and VA.^49^ In addition, recently published and ongoing studies suggest a highly beneficial direct effect of local anaesthetics on carcinoma.^50,51,52^ Intraoperative intravenous lidocaine infusion has been associated with reduced intraoperative opioid use and improved overall survival in patients undergoing pancreatic cancer surgery.^53^

Opioids have been shown to have an unfavourable effect on tumour growth *in vitro*.^54,55,56^ Several clinical studies have been published and show a complex relationship that depends on many factors, such as the type of opioid, the amount of opioid administered and adjuvants. The results of the studies are highly controversial but tend to favour a harmful effect of opioids.^44,55^ The different findings on the cancer risk of opioids are a line of research that needs to be pursued as they have major implications for clinical practise given the importance of opioid use in anaesthetic practice and pain management.

The exception is tramadol, which is supposed to protect the body against metastases. It does not inhibit the immune system like other opioids.^57^ Unfortunately, tramadol is rarely used in oncology due to its weak analgesic effect and unpleasant side effects at higher doses.

There are also some studies in the field of anaesthetic adjuvants such as dexmedetomidine and clonidine. *In vitro* results indicated their unfavourable effect on the growth and spread of cancer, but clinical studies have not confirmed it.^58,59,60^ Studies have shown that dexmedetomidine has a positive effect on patients anaesthetised with sevoflurane, possibly because it reduces neuroinflammation.^61^ However, further studies are needed in this area.

However, there are also some studies on the use of other agents. Nonsteroidal anti-inflammatory drugs have potential anticancer effects.^62^ Beta-blockers affect cancer growth and spread by reducing the sympathetic stress response.^63^ Dexamethasone reduces inflammation and the immune response by inhibiting NK cells and thus has an unfavourable effect, but low antiemetic doses are not thought to increase cancer growth and spread.^64^ Oxygen causes ROS synthesis and oxidative stress and can induce various degrees of partial to complete transformation from epithelium to mesenchyme in cancer cells. Even if the primary tumours are surgically removed, the effects of hyperoxia on micrometastases and circulating cancer cells may promote cancer progression or recurrence. Therefore, it is necessary to use the lowest sufficient concentrations of oxygen.^65^

## Conclusions

Recent studies have shown that anaesthesia may play an important role in the growth and spread of cancer. Volatile anaesthetics have proinflammatory effects and can therefore accelerate metastasis. Propofol has an anti-inflammatory and antioxidant effect, causes less neuroinflammation and may have an antitumour effect. Regional anaesthesia plays an important role in reducing the likelihood of metastasis after surgery, as local anaesthetics have a protective effect on cancer recurrence. Opioids, except for tramadol, can accelerate cancer growth and spread and should be avoided or reduced perioperatively. Dexmedetomidine has no effect on the tumour, although it modulates inflammation.

In summary, there are still no clear answers to questions about the carcinogenicity of agents and techniques used during anaesthesia. The field needs further research.
